# MiR-566 mediates cell migration and invasion in colon cancer cells by direct targeting of *PSKH1*

**DOI:** 10.1186/s12935-019-1053-1

**Published:** 2019-12-11

**Authors:** Ying Zhang, Siqi Zhang, Jian Yin, Ruisi Xu

**Affiliations:** 1 Endoscopy Center, China-Japan Union Hospital of Jilin University, No. 126 Sendai Street, Changchun, 130033 Jilin China; 2 Department of Nephrology, China-Japan Union Hospital of Jilin University, Changchun, 130033 Jilin China; 3 Department of Vascular Surgery, China-Japan Union Hospital of Jilin University, Changchun, 130033 Jilin China

**Keywords:** Colorectal cancer, miR-566, PSKH1, Invasion, Migration

## Abstract

**Background:**

Colorectal cancer (CRC), a common malignancy worldwide, and microRNAs (miRs) have been suggested to play roles in the disease. MiR-566 expression has been shown to be reduced in CRC, but its functions and mechanisms are still unclear.

**Methods:**

Cell viability was assessed by using the CellTiter 96 AQueous One Solution Cell Proliferation kit. Cell proliferation was measured with MTT assay. Cell metastasis were measured by transwell assay. Luciferase reporter assays was used to confirm the target of MiR-566. PSKH1 expression was measured by RT-PCR and western blot.

**Results:**

In the present study, we first observed that miR-566 was expressed in several CRC cell lines (SW480, SW620, LoVo, HT29 and Caco-2) at low levels compared to control colon epithelial cell lines (FHC). Further study showed that miR-566 overexpression suppressed cell survival and impeded cell proliferation, whereas inhibition of its expression enhanced cell survival and proliferation. Transwell assays showed that cell invasion and migration were reduced in cells overexpressing miR-566 and increased in those with inhibition of miR-566. Further analysis confirmed that *PSKH1* is a target of miR-566. MiR-566 overexpression significantly inhibited PSKH1 expression and reintroduction of PSKH1 partially reversed the effects of miR-566 on CRC cell growth and metastasis in SW480 and Caco-2 cells.

**Conclusions:**

Taken together, the data show that CRC cell growth and metastasis can be significantly suppressed by miR-566 through targeting *PSKH1*.

## Background

Colorectal cancer (CRC), the most prevalent malignancy, is a complex polygenetic disease and the main cause of death from cancer worldwide [1, 2]. CRC-associated morbidity is increasing annually, and in 2008, there were approximately 120,0000 new CRC cases globally and more than 60,0000 deaths [3]. Death is caused by progression and metastasis of the cancer, and previous research implicates several underlying mechanisms in cancer metastasis, including tumor cell invasion, adhesion, chemotaxis, epithelial–mesenchymal transition and tumor cell growth [3, 4]. In recent years, the life expectancy of patients with CRC has improved because of the advances in screening and treatment; however, the prognosis remains poor in patients with metastatic cancer [5]. Thus, understanding how cancer metastasis proceeds and finding a way to block this process would be beneficial in developing cancer treatments.

MicroRNAs (miRNAs), a family of single-stranded, noncoding, short RNAs, have been shown to be involved in CRC metastasis [6–8]. miRNAs play inhibitory roles in the function of their target genes, with miRNAs binding to the 3ʹ untranslated region (3ʹUTR) of target genes and then inhibiting gene translation or degrading the target mRNA [9]. Prior evidence suggests that miRNA may be used as a cancer therapy because it can interact with many target genes and signaling pathways that promote cancer cell death [10]. Li et al. [11] showed that miR-433 plays roles in inhibiting growth and promoting apoptosis through mediation of its target gene MACC1. In addition, Qin et al. [12] suggested that mir-106a regulates the PTEN/PI3K/AKT pathway and is associated with colon cancer cell growth. Multiple studies have implicated miRNAs play roles in colon cancer cell migration and invasion [13, 14]. miR-566 has been shown to be decreased in CRC; however, its functions and mechanisms remain unknown [15]. The present study found that miR-566 has low expression in colon cancer cells and plays roles in cancer cell growth and metastasis.

Kim and his colleagues [16] found that three kinase genes, *PHKG2*, *TLK2*, and *PSKH1*, were all expressed in metastatic CRC at high level, and their overexpression may be a potential biomarker of cetuximab plus irinotecan induced—wild-type KRAS CRC. PSKH1, an autophosphorylating human protein serine kinase, has 424 amino acids and is localized to the Golgi apparatus and speckle structures within the nucleus [17, 18]. In the current study, we confirmed that *PSKH1* is a direct target of miR-566, and its expression was inhibited by miR-566 overexpression; moreover, our study indicated that PSKH1 is involved in the function of miR-566 in CRC cell proliferation, migration, and invasion.

## Materials and methods

### Cell culture and transfection

The human colon cancer cell lines SW480, SW620, LoVo, Caco-2 and HT29 and human normal colon epithelial cell lines (FHC) were purchased from ATCC (Manassas, VA, USA). All cells were maintained in DMEM added with 100 µg/mL streptomycin, fetal bovine serum, 100 U/mL penicillin. Cells were cultured in a 5% CO_2_ atmosphere at 37 °C.

For transfection, micro-RNAs [19] used in this study were all obtained from GenePharma (Shanghai, China). Lipofectamine^®^ RNAiMAX Transfection Reagent (Invitrogen, Carlsbad, CA) were used to transfected miR-566 mimic (F: 5′-GGGCGCCUGUGAUCCCAAC-3′; R: 5′-UGGGAUCACAGGCGCCCUU-3′), mimic control (NC: F: 5′-UUCUCCGAACGUGUCACGUTT-3′; R: 5′-ACGUGACACGUUCGGAGAATT-3′), miR-566 inhibitor (5′-GUUGGGAUCACAGGCGCCC-3′) and inhibitor control (anti-NC: 5′-CAGUACUUUUGUGUAGUACAA-3′) into human colon cancer cells. For further studies of the mechanism, SW480 and Caco-2 cells were co-transfected with miR-566 mimic and pcDNA3.1-PSKH1 vector and cultured for 48 h.

### Cell survival

SW480, SW620 or Caco-2 cell viability was assessed by using the CellTiter 96 AQueous One Solution Cell Proliferation kit (Promega, Madison, WI, USA) following the manufacturer’s instructions.

### Cell proliferation

SW480, SW620 or Caco-2 cell proliferation was measured with MTT assay. Cells were transfected with miR-566 mimic, NC, miR-566 inhibitor, and anti-NC for 48 h, and MTT (20 μL, 5 mg/mL) was added to cells for another 4 h. Cell proliferation was determined by quantification of the absorbance at 490 nm by using a microplate reader.

### Transwell assay

SW480 or SW620 or Caco-2 cell metastasis were measured by transwell assay. SW480 or SW620 cells at a density of 1 × 10^5^ were plated into the upper transwell chamber. For the invasion assay, the upper transwell chamber was coated with Matrigel, and for the migration assay, the chamber was uncoated. The lower transwell chamber contained the chemoattractant (10% serum). The invading or migrating cells were stained with 0.1% crystal violet and quantified by using a microscope.

### Quantitative real-time PCR

Cells were transfected as already described. Total RNA was isolated from SW480 and SW620 cells using TRIzol. For miR-566 detection, 1 μg of total RNA was reverse-transcribed into cDNA using the miScript Reverse Transcription kit (Qiagen, Hilden, Germany). RT-PCR was performed with a miScript SYBR-Green PCR kit (Qiagen). Expression levels were normalized to U6.

### Western blot

Total protein was extracted from colon cancer cells with RIPA lysis buffer (1% Nonidet P-40, 50 mM Tris (pH7.4), 0.5% deoxycholic acid, 100 mM NaCl, 10 mg/mL aprotinin, 1 mM phenylmethylsulfonylfluoride, 0.1% sodium dodecyl sulfate, and 10 mg/mL leupeptin) [20]. Equal amounts of protein (40 μg/lane) were separated by 10% SDS-PAGE and transferred onto PVDF membranes. Rabbit polyclonal anti-PSKH1 antibody, mouse monoclonal anti-E-cadherin antibody, mouse monoclonal anti-vimentin antibody, and rabbit polyclonal anti-N-cadherin antibody (Abcam, Cambridge, MA, USA) were used to probe the proteins. The signals were measured by using an ECL detection system. β-actin (Abcam, Cambridge, MA, USA) was used as a loading reference for data analysis.

### Luciferase reporter assays

Luciferase reporter assays were performed following previously described methods [21]. pGL3 control vectors (Invitrogen) containing the wild-type and mutant-type reporter 3ʹ-UTR of PSKH1 (Wt-PSKH1 and Mut-PSKH1) were synthesized. SW480 cells were co-transfected with Wt-PSKH1 and miR-566 mimic or miR-NC or with Mut-PSKH1 and miR-566 mimic or miR-NC for 48 h. Luciferase activity was assayed by using the Dual luciferase assay kit (Promega).

### RNA immunoprecipitation assay

RNA immunoprecipitation was implemented using an EZ-Magna RIP RNA-Binding Protein Immunoprecipitation Kit (Millipore, Billerica, MA, USA) according to the manufacturer’s instruction. SW480 cells transfected with anti-NC or miR-566 inhibitor were lysed into RNA immunoprecipitation lysis buffer. Cell lysate was incubated with RNA immunoprecipitation buffer containing magnetic beads conjugated with human anti-Ago2 antibody (1:50 dilution) or negative control IgG. The precipitated RNAs were subjected to real-time PCR to measure the RNA levels of miR-566 and PSKH1.

### Statistical analysis

Data in this study are presented as mean ± SD. SPSS 19.0 (IBM SPSS, Armonk, NY, USA) was used to statistical analyses. Student’s *t*-test was used to detect differences between two groups. One-way ANOVA followed by LSD test was used to detect differences between three or more groups. Differences were considered significant at *p* < 0.05. All data were performed in triplicate.

## Results

### MiR-566 overexpression inhibits CRC cell survival and proliferation

**Fig. 1:**
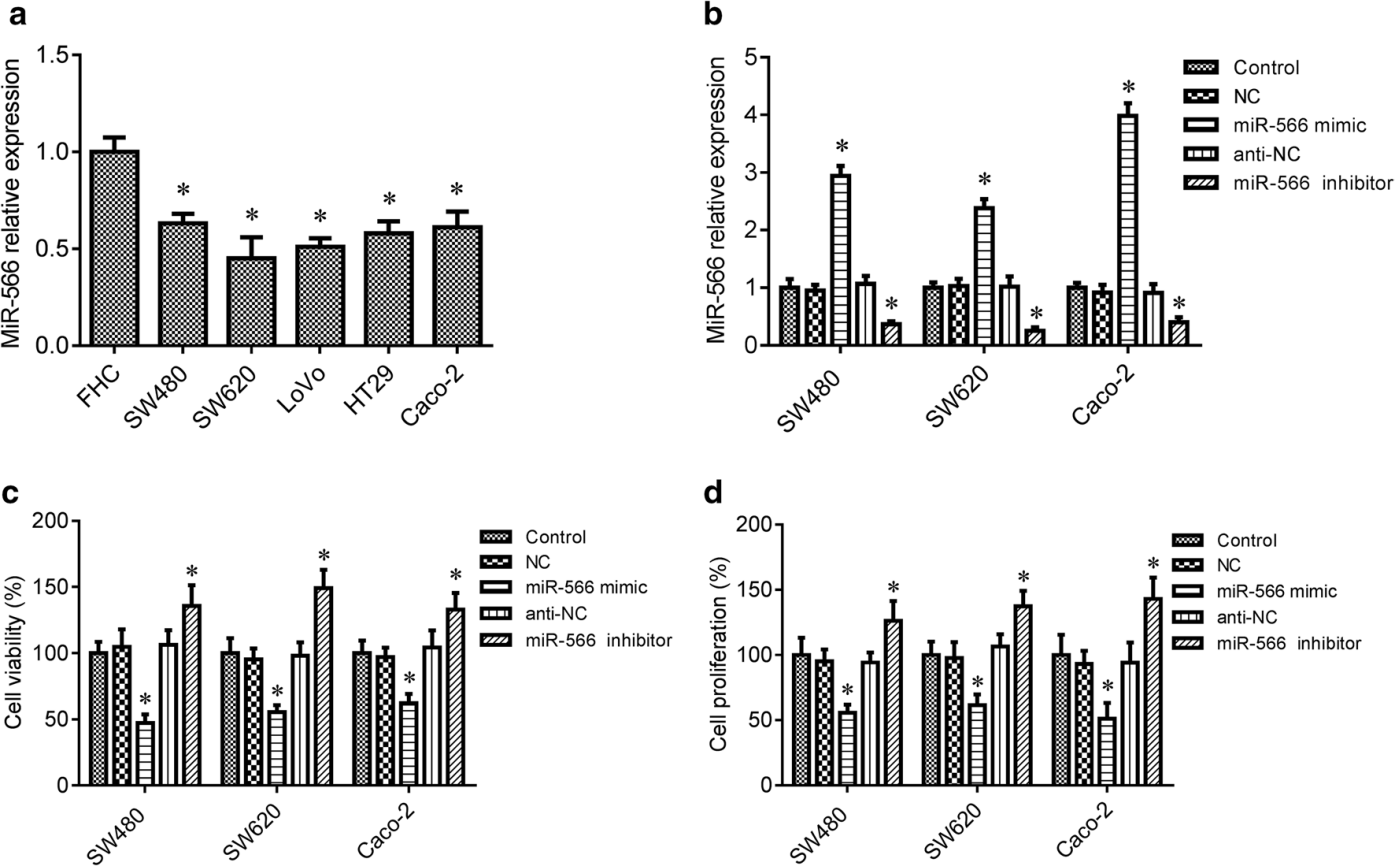
miR-566 overexpression suppressed CRC cell survival and proliferation. **a** RT-PCR was used to measure the expression of miR-566 in CRC cell line SW480, SW620, LoVo, Caco-2 and HT29, and the control colon epithelial cell line FHC. All experiments were performed in triplicate. **b** RT-PCR was used to measure the expression of miR-566. **c** CellTiter 96 AQueous One Solution Cell Proliferation kit was used to determine cell survival. **d** Cell proliferation was determined by MTT assay. SW480, SW620 and Caco-2 cells were transfected with miR-566 mimic, the mimic control (NC), miR-566 inhibitor, or inhibitor control (anti-NC) for 48 h. All experiments were performed in triplicate. *p < 0.05 vs. control

Prior research suggested that miR-566 is decreased in CRC [15]. In the in vitro cell assay, we measured miR-566 levels in the five CRC cell lines SW480, SW620, LoVo, Caco-2 and HT29 and the control colon epithelial cell lines FHC by RT-PCR assay. The results showed that miR-566 was significantly lower in SW480, SW620, LoVo, Caco-2 and HT29 cells, when compared to the control FHC cells (Fig. 1a, *p* < 0.05). To confirm the function of miR-566 in CRC cells, loss-of-function and gain-of-function experiments were conducted for miR-566 in SW480, SW620 and Caco-2 cells through transfecting cells with miR-566 mimic and inhibitor, respectively. As shown in Fig. 1b, the miR-566 mimic markedly increased the level of miR-566 in SW480, SW620 and Caco-2 cells, whereas the miR-566 inhibitor decreased the levels of miR-566 in of the three cell lines (*p* < 0.05). Further study indicated that miR-566 overexpression inhibited cell survival and proliferation in SW480, SW620 and Caco-2 cells compared to the control group (*p* < 0.05), whereas inhibition of miR-566 expression by using miR-566 inhibitor promoted survival and proliferation of these cells (Fig. 1c, d, *p* < 0.05).

### MiR-566 overexpression inhibits CRC cell migration and invasion

**Fig. 2:**
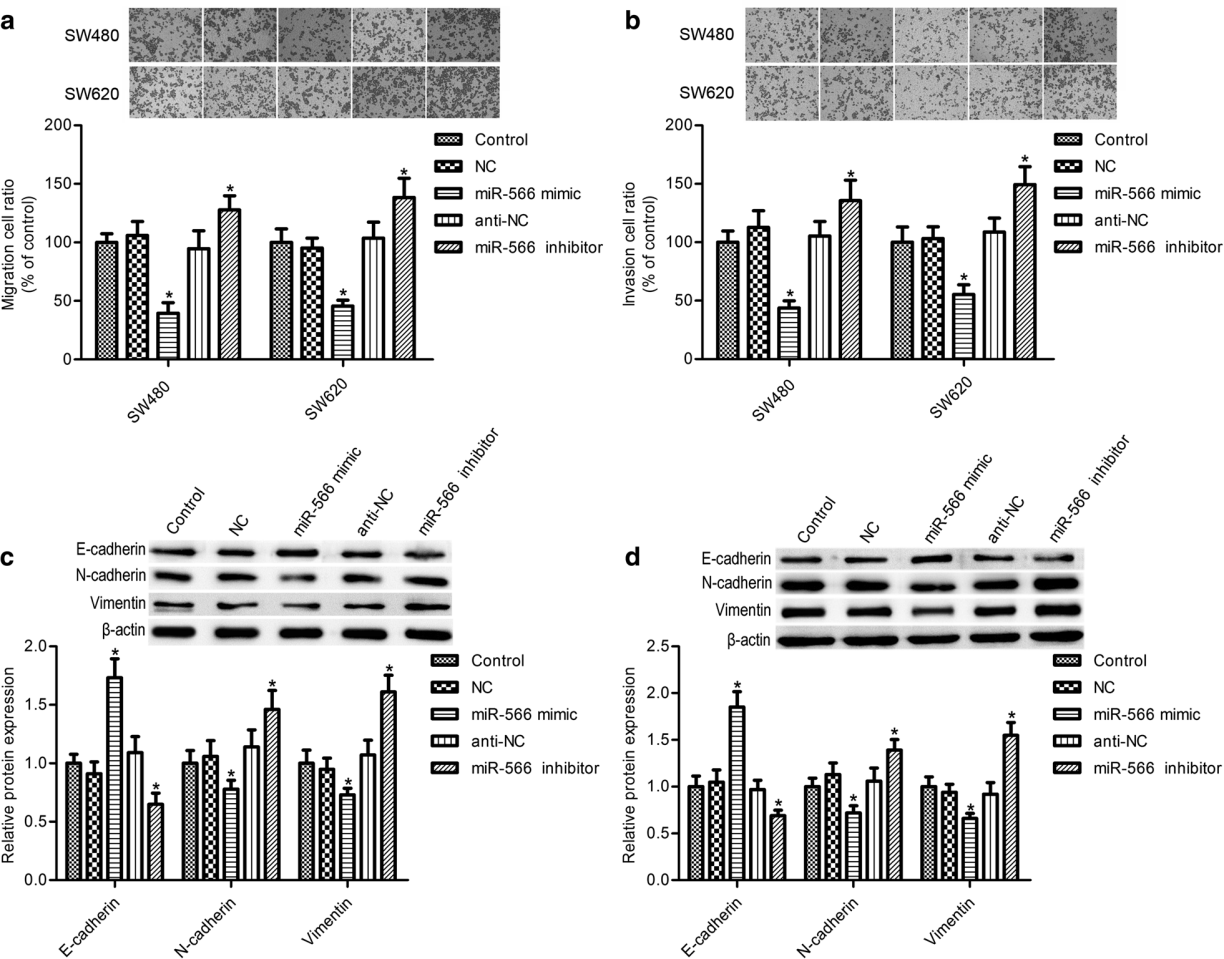
miR-566 overexpression suppressed CRC cell migration and invasion. **a**, **b** SW480 and SW620 cell migration and invasion were assayed by transwell assay. Expression of epithelial–mesenchymal transition markers E-cadherin, vimentin, and N-cadherin in SW480 cells (**c**) and SW620 cells (**d**) were measured by western blot assay. SW480 and SW620 cells were transfected with miR-566 mimic, the mimic control (NC), miR-566 inhibitor, or inhibitor control (anti-NC) for 48 h. All experiments were performed in triplicate. *p < 0.05 vs. control

As shown in Fig. 2a, b, miR-566 mimic transfection markedly suppressed SW480 and SW620 cell migration and invasion (*p *< 0.05), while miR-566 inhibition significantly increased them (*p *< 0.05). Epithelial–mesenchymal transition (EMT) plays important roles in cell migration and invasion. We found that miR-566 overexpression markedly elevated the epithelial marker E-cadherin expression and impeded the mesenchymal markers vimentin and N-cadherin levels, whereas its inhibition had opposite effects on E-cadherin, vimentin, and N-cadherin expression (Fig. 2c, d, *p* < 0.05).

### PSKH1 is a target of miR-566

**Fig. 3:**
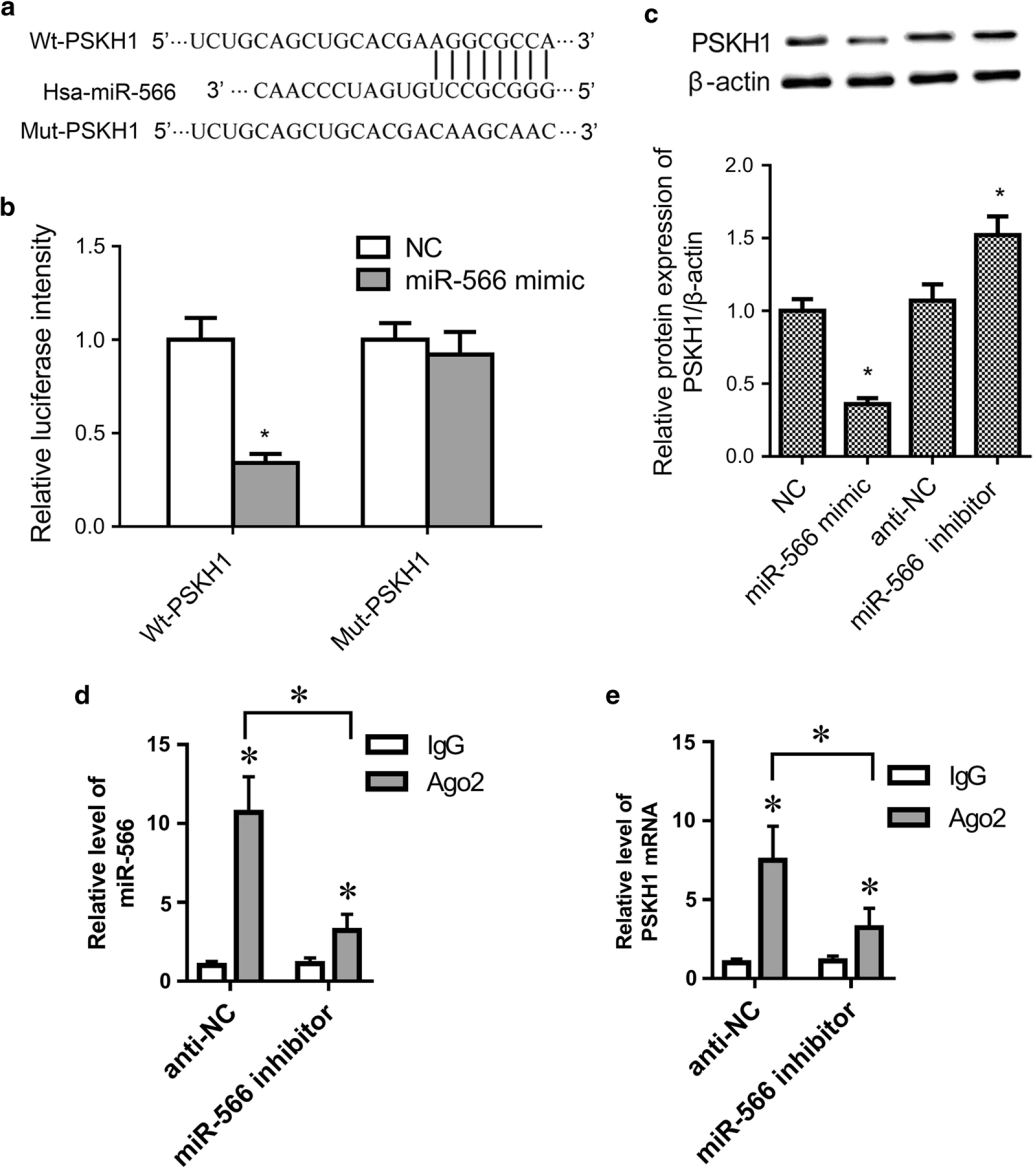
miR-566 suppressed *PSKH1* expression in SW480 cells. **a** Computational analysis suggested putative binding sites between miR-566 and 3ʹ-UTR of human PSKH1. **b** SW480 cells were co-transfected with miR-566 mimic and wild-type PSKH1 3ʹ-UTR (Wt-PSKH1) or mutant PSKH1 3ʹ-UTR (Mut-PSKH1). Luciferase reporter assay was used to show PSKH1 is a direct target of miR-566. **c** PSKH1 protein expression was determined by Western blot assay. **d**, **e** RNA immunoprecipitation assay was performed using anti-Ago2 in SW480 cells transfected with anti-NC or miR-566 inhibitor. MiR-566 and 3′-UTR of PSKH1 mRNA levels in the anti-Ago2 or anti-IgG immunoprecipitated products were measured by RT-PCR. All experiments were performed in triplicate. *p < 0.05 vs. control

Computational analysis suggested putative binding sites between miR-566 and 3ʹ-UTR of human *PSKH1*, as shown in Fig. 3a. To confirm whether *PSKH1* is a target of miR-566, we used luciferase reporter assays. Compared to the NC group, the relative luciferase activity was significantly decreased in miR-566 mimic and wild-type *PSKH1* 3ʹ-UTR (Wt-*PSKH1*) co-transfected cells (*p* < 0.05), while no significant differences were found between the miR-566 mimic and mutant *PSKH1* 3ʹ-UTR (Mut-*PSKH1*) co-transfected group and NC group (Fig. 3b, *p* > 0.05). Western blot assay further showed that PSKH1 expression was inhibited by miR-566 overexpression (Fig. 3c, *p* < 0.05). RNA immunoprecipitation assay was performed using anti-Ago2 in SW480 cells transfected with anti-NC or miR-566 inhibitor. The result showed that both miR-566 and 3′-UTR of PSKH1 mRNA were detected in the anti-Ago2 immunoprecipitated products. MiR-566 inhibitor transfection significantly inhibited miR-566 and 3′-UTR of PSKH1 mRNA levels in the anti-Ago2 immunoprecipitated products (Fig. 3d, e, *p* < 0.05).

### Reintroduction of PSKH1 reversed the effects of miR-566 on CRC cell migration and invasion

**Fig. 4:**
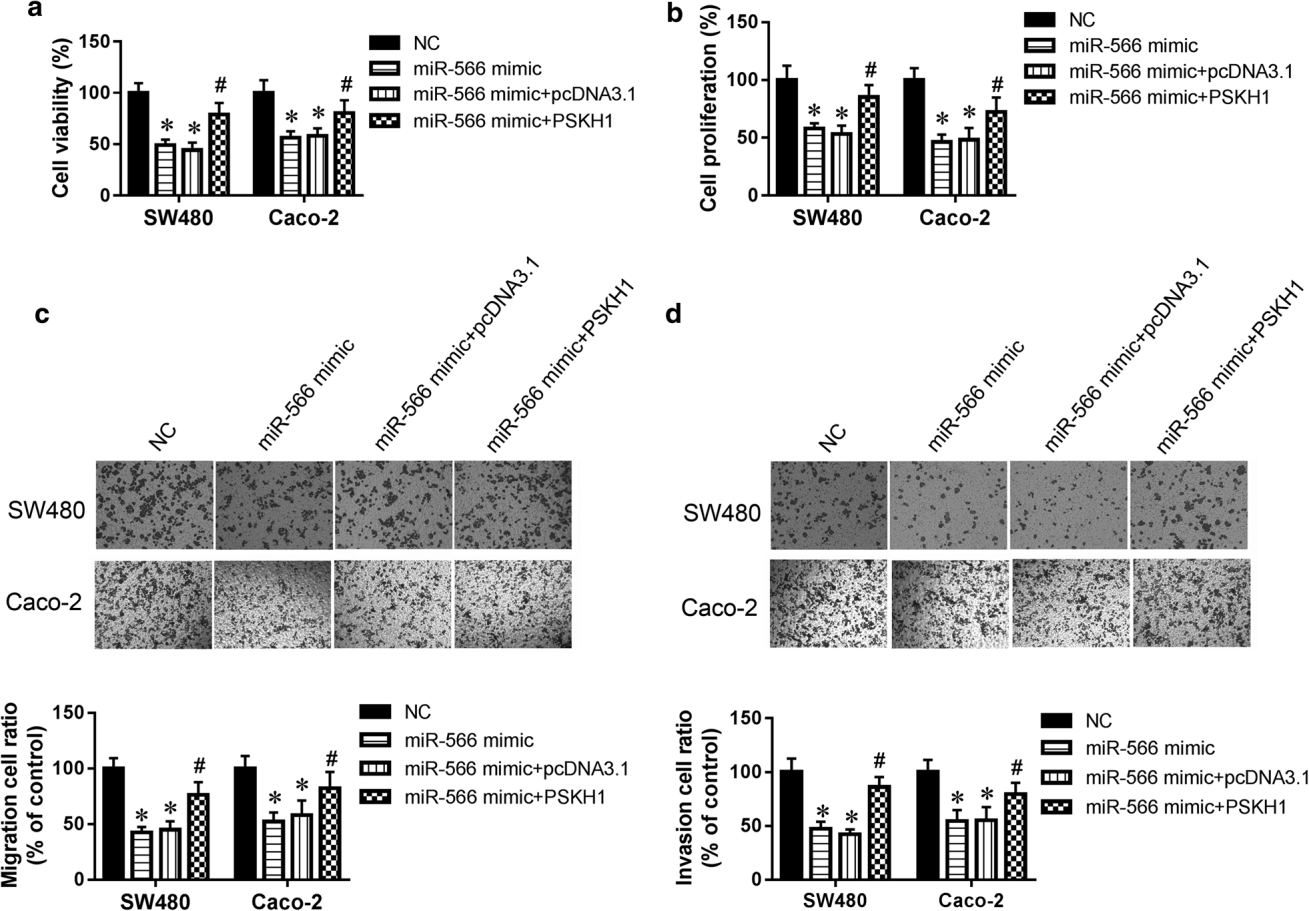
PSKH1 was involved in the effect of miR-566 on CRC cell migration and invasion. **a** CellTiter 96 AQueous One Solution Cell Proliferation kit was used to determine survival of SW480 and Caco-2 cells. **b** Proliferation of SW480 and Caco-2 cells was determined by MTT assay. **c** Representative images and quantitative results for migration assay of SW480 and Caco-2 cells by transwell. **d** Representative images and quantitative results for invasion assay of SW480 and Caco-2 by transwell. All experiments were performed in triplicate. *p < 0.05 vs. NC, ^#^*p *< 0.05 vs. miR-566 mimic group

To further validate PSKH1 as a mediator of the effects of miR-566 on CRC growth, migration, and invasion, SW480 cells were co-transfected with miR-566 mimic and pCMV-PSKH1 vector for 48 h. The results suggested that the PSKH1 overexpression vector reversed the miR-566-mediated inhibition of SW480 cell survival (Fig. 4a). Furthermore, the PSKH1 overexpression vector increased SW480 cell proliferation, promote cell migration and invasion compared with the miR-566 overexpression group (Fig. 4b–d). It was reported that both SW480 and SW620 cell lines have TP53 and KRAS mutations, but the Caco-2 cells has only one mutation in TP53 [22]. So, Caco-2 cells were also employed in our study. The data from Caco-2 cells were in accordance with SW480 cells (Fig. 4a–d). Above results indicate that the effects of miR-566 in CRC cell growth, migration, and invasion depend on *PSKH1* inhibition.

## Discussion

Colorectal cancer is ranks the third diagnosed among cancer types worldwide, and it is the main cause of mortality from cancer [23]. Cancer metastasis plays roles in the advance of cancer, and in this study, we first confirmed that miR-566 is involved in colon cancer metastasis through targeting *PSKH1*.

MiRNAs are single-stranded, 20- to 23-nucleotide-long, noncoding RNAs that are involved in the regulation of carcinogenic pathways [24, 25]. Compared with mRNAs, miRNAs are less likely to be degraded by Ranse because of their stem-loop and small-size structure [26]; thus, this conserved molecule is easy to detect in tissue and body fluids, including saliva, urine and blood [27]. miRNAs have been well-documented to act as tumor biomarkers, and they have been shown to be associated with the development of cancer, including colorectal cancer [28, 29]. Prior reports indicated that miRNAs are potential tumor suppressor or oncogenes by regulating many cell processes including the cell cycle, proliferation, apoptosis, and invasion. Pan et al. [19] reported that the oncogene miR-566 is a potential biomarker of renal cell carcinoma prognosis.

MiR-566 was aberrantly expressed in many tumor types, such as lung adenocarcinoma [30], glioblastoma [31, 32], and colon cancer [15]. Consistent with these previous reports, our study demonstrated that miR-566 had a low expression level in several CRC cell lines (SW480, SW620, LoVo, Caco-2 and HT29) compared to the control colon epithelial cell line (FHC). In addition, Xiao et al. [32] previously found that miR-566 inhibition impeded glioblastoma migratory and invasive abilities through decreased VEGF expression and increased VHL expression. We also observed that miR-566 overexpression suppressed survival, proliferation, migration, and invasion in SW480 and SW620 cells.

Protein kinases play important roles in many cellular processes, such as cell cycle progression, apoptosis, cell movement, metabolism, cytoskeletal rearrangement, transcription, and cell differentiation [33, 34]. PSKH1 is an autophosphorylating human protein serine kinase with 424 amino acids that has been suggested to play structural and regulatory roles in cells and to be involved in the maintenance of the Golgi apparatus [35]. Kim et al. [16] suggested that PSKH1 is highly expressed in patients with colon cancer. To search the specific potential target gene for miR-566, an online computational algorithm TargetScan (http://www.targetscan.org) was used. *PSKH1* was a potential target gene for miR-566, and potential binding sites were found in its 3ʹ-UTR region. Our study first demonstrated that *PSKH1* is a direct target of miR-566, and its expression was influenced by the level of miR-566.

## Conclusion

In conclusion, the current study demonstrated that miR-566 is decreased in colon cancer cells, and overexpression of miR-566 inhibited cancer cell growth and metastasis. In addition, we found that *PSKH1* is a target of miR-566, and reintroduction of PSKH1 expression reversed the effects of miR-566 on cancer cell growth and metastasis.

## Data Availability

All data generated or analyzed during this study are included in this published article.
